# Overweight and obesity prevalence among Cree youth of Eeyou Istchee according to three body mass index classification systems

**DOI:** 10.1186/s12887-017-0951-4

**Published:** 2017-11-22

**Authors:** Audray St-Jean, Salma Meziou, Pierre Ayotte, Michel Lucas

**Affiliations:** 1 Population Health and Optimal Health Practices Research Unit, CHU de Québec – Université Laval, Hôpital du Saint-Sacrement, 1050 Chemin Sainte-Foy, Québec, QC G1S 4L8 Canada; 2 Institut National de Santé Publique du Québec (INSPQ), 945 Avenue Wolfe, Québec, QC G1V 5B3 Canada; 3 Department of Social and Preventive Medicine, Faculty of Medecine, Université Laval, 1050 Avenue de la Médecine, Local 2428, Québec, QC G1V 06A Canada

**Keywords:** Body mass index, Youth, Obesity, Overweight, Eeeyou Istchee Cree

## Abstract

**Background:**

Little is known about the suitability of three commonly used body mass index (BMI) classification systems for Indigenous youth. We estimated overweight and obesity prevalence among Cree youth of Eeyou Istchee according to three BMI classification systems, assessed the level of agreement between them, and evaluated their accuracy through body fat and cardiometabolic risk factors.

**Methods:**

Data on 288 youth (aged 8–17 years) were collected. Overweight and obesity prevalence were estimated with Centers for Disease Control and Prevention (CDC), International Obesity Task Force (IOTF) and World Health Organization (WHO) criteria. Agreement was measured with weighted kappa (κw). Associations with body fat and cardiometabolic risk factors were evaluated by analysis of variance.

**Results:**

Obesity prevalence was 42.7% with IOTF, 47.2% with CDC, and 49.3% with WHO criteria. Agreement was almost perfect between IOTF and CDC (κw = 0.93), IOTF and WHO (κw = 0.91), and WHO and CDC (κw = 0.94). Means of body fat and cardiometabolic risk factors were significantly higher (*P*_trend_ < 0.001) from normal weight to obesity, regardless of the system used. Youth considered overweight by IOTF but obese by CDC or WHO exhibited less severe clinical obesity.

**Conclusions:**

IOTF seems to be more accurate in identifying obesity in Cree youth.

## Background

Overweight and obesity are defined as abnormal or excessive fat accumulation that may impair health [1]. Associations are well-documented for overweight and obesity in childhood and adolescence with cardiovascular and metabolic complications as well as premature mortality in adulthood [2, 3]. The high prevalence of obesity among youth has become a major public health issue [4]. In Canada, obesity is more pervasive among Indigenous than non-Indigenous youth [5, 6].

Body mass index (BMI) is the most common method of assessing weight status and health risks in youth. Because BMI varies with growth and maturation during childhood and adolescence, age- and sex-specific cut-off points are needed for appropriate overweight and obesity classification. Three BMI classification systems are commonly used to study youth, with cut-off values published by the International Obesity Task Force (IOTF) [7], the Centers for Disease Control and Prevention (CDC) [8], and the World Health Organization (WHO) [9]. IOTF cut-offs are derived from six large surveys conducted in Brazil, UK, Hong Kong, the Netherlands, Singapore, and USA. CDC growth references are based on data from five nationally representative surveys of American youth. WHO growth curves are drawn from the WHO Multicentre Growth Reference Study conducted in six countries (Brazil, Ghana, India, Norway, Oman, and USA).

Inconsistent overweight and obesity prevalence estimation according to these classification systems poses challenges. In the same population, estimates tend to be the lowest for IOTF whereas WHO cut-offs appear to be the highest [10–13]. Our team previously studied prevalence estimates of overweight and obesity among Inuit youth according to IOTF, CDC and WHO criteria [14]. These BMI classification systems were based on populations that did not include Indigenous youth. The Cree are a unique cultural and ethnic group. Therefore, it is particularly interesting to verify the suitability of such BMI systems in this population.

The present study estimated overweight and obesity prevalence among Cree youth of Eeyou Istchee, northern Quebec according to three BMI classification systems, assessed the level of agreement between these classification systems, and evaluated their accuracy with body fat percentage and cardiometabolic risk factors as surrogates of obesity-related outcomes.

## Methods

### Study design and population

Data were sourced from the cross-sectional “*Nituuchischaayihtitaau Aschii*: A Multi-Community Environment-and-Health Study in Eeyou Istchee*”*, a collaboration of the Cree Board of Health and Social Services of James Bay (CBHSSJB) with Laval, McGill and McMaster Universities. The study design has been described previously [15]. Briefly, a random sample of participants was recruited from seven communities of Eeyou Istchee (latitude > 49.6° N). Data were collected during the spring and/or summer of 2005, 2007, 2008 and 2009. Participants were advised to fast overnight and, during the next day’s appointment, a research nurse measured anthropometric data and collected venous blood samples which were kept frozen at −80 °C and transported to the CHUQ Research Centre, Québec (Canada), for biological analysis. Inclusion criteria for the present analysis were age 8–17 years and blood samples collected under fasting conditions (≥ 8 h). The study population also included participants aged between 0 and 7 years old. However, anthropometric and clinical measurements were not assessed in this age group. Two of the initial 290 participants selected were excluded because of missing BMI data, leaving 288 participants for analysis.

Participation was voluntary, and written informed consent was given by one of the child’s parents or guardian. Ethics approval was obtained from all participating institutions.

### Anthropometric data

Weight without shoes was measured with a bioelectrical impedance scale (Tanita Corp., Arlington Heights, IL, USA). Height without shoes was quantified using a measuring tape with patients standing barefoot on a hard surface. Waist circumference (WC) was assessed at the end of exhalation by tape located midway between the lower margin of the last floating rib and the top of the iliac crest [1]. Height was recorded to the nearest cm, and WC, to the nearest 0.5 cm. BMI was calculated by dividing weight (kg) by squared height (m^2^).

Weight status was defined according to 2005 IOTF [7], 2000 CDC [8], and 2007 WHO criteria [9]. IOTF cut-off values are age- and sex-specific extrapolations of adult overweight (BMI ≥ 25 kg/m^2^) and obesity (BMI ≥ 30 kg/m^2^) definitions at 18 years. CDC BMI age- and sex-specific growth references classify overweight as 85^th^ percentile ≤ BMI < 95^th^ percentile, and obesity, as BMI ≥ 95^th^ percentile. WHO BMI-for-age categorizes overweight as BMI > +1 standard deviation above the WHO growth standard median while BMI > +2 standard deviations is considered as obesity. Participants who were neither overweight nor obese were defined as normal weight. BMI z-scores were calculated by the CDC SAS program [16]. Body fat percentage (%) was assessed by bioelectrical impedance analyzer (Tanita TBF-300, GHT Canada, Laval, QC, Canada).

### Cardiometabolic risk factors

Systolic (SBP) and diastolic blood pressure (DBP) were measured according to Canadian Hypertensive Education Program recommendations [17]. Fasting plasma glucose was quantified by spectrophotometric assay (Vitros 950 Chemistry Station, Ortho-Clinical Diagnostics, Raritan, NJ, USA), and fasting plasma insulin, by immunoassay with chemiluminescent detection (Advia Centaur, Siemens, Washington, DC, USA). Homeostatic model assessment 2 of insulin resistance (HOMA2-IR) was calculated from fasting plasma glucose and insulin levels [18]. Triglycerides (TG) and high-density lipoprotein cholesterol (HDL-C) were evaluated by enzymatic methods (Vitros 950 Chemistry Station, Ortho-Clinical Diagnostics). TG/HDL-C ratio was ascertained by dividing TG by HDL-C concentrations. Lipids were not assessed in participants aged 8 to 14 years during the 2005 (1 community) and 2007 (2 communities) surveys, which corresponded to missing values for 91 participants.

### Statistical analyses

The characteristics of study participants per gender were compared by *t*-tests and reported as arithmetic means ± standard deviations. Overweight and obesity prevalence estimates (%) according to the three BMI classification systems were presented graphically, and differences between prevalence rates were compared by chi-square tests. Agreements between BMI classification systems were evaluated by weighted kappa (κw) coefficients according to Landis and Koch’s guiding principles (19). κw coefficients between 0 and 0.20 are considered as slight, 0.21–0.40 as fair, 0.41–0.60 as moderate, 0.61–0.80 as substantial, and 0.81–1.00 as almost perfect [19]. Analysis of variance was used to investigate whether means of body fat and cardiometabolic risk factors were different according to weight status. Tests for trend were assessed by assigning the median BMI value to each weight status category and modelling this value as a continuous variable using the contrast statement of the SAS PROC GLM procedure. Mean differences in body fat and cardiometabolic risk factors according to agreement and or non-agreement between weight status based on IOTF/CDC and IOTF/WHO classification systems were calculated. All statistical analyses were performed with SAS software (version 9.4, SAS Institute Inc., Cary, NC, USA). Two-sided *P* < 0.05 values were considered to be statistically significant.

## Results

Characteristics of study participants from the seven Cree communities are presented by gender in Table 1. Mean age was 12.4 years (range 8–17 years), 50.3% were girls, and mean BMI z-score was 1.3. Differences between the two genders were statistically significant for numerous variables. Girls had significantly higher body fat, fasting plasma insulin and HOMA2-IR score than boys. Weight, height and SBP were significantly lower among girls compared to boys.

**Table 1:** Characteristics of study participants (8–17 years) from Eeyou Istchee communities of northern Quebec, Canada, 2005–2009

	Total (*n* = 288)	Boys (*n* = 143)	Girls (*n* = 145)	*P*
Age, years	12.4 ± 2.7	12.7 ± 2.7	12.1 ± 2.8	0.10
Anthropometric data				
Height, cm	159 ± 13.6	162 ± 14.7	156 ± 11.7	< 0.001
Weight, kg	64.7 ± 22.6	67.5 ± 23.7	62.0 ± 21.2	0.04
WC, cm	88.3 ± 15.7	89.9 ± 16.1	86.7 ± 15.2	0.08
BMI, kg/m^2^	25.1 ± 6.1	25.1 ± 5.9	25.0 ± 6.2	0.93
BMI z-score	1.3 ± 1.0	1.3 ± 1.0	1.3 ± 1.0	0.46
Body fat, %	32.5 ± 10.9	29.0 ± 11.4	36.1 ± 9.1	< 0.001
Cardiometabolic risk factors				
SBP, mm Hg	105 ± 12.1	107 ± 12.8	103 ± 11.0	0.01
DBP, mm Hg	62 ± 10.6	63 ± 11.1	61 ± 10.1	0.09
Fasting plasma glucose, mmol/L	5.1 ± 0.44	5.1 ± 0.42	5.0 ± 0.46	0.05
Fasting plasma insulin, pmol/L	145 ± 109	129 ± 95.7	160 ± 120	0.02
HOMA2-IR	2.5 ± 1.7	2.3 ± 1.4	2.8 ± 1.8	0.008
TG, mmol/L	1.0 ± 0.51	0.90 ± 0.44	1.0 ± 0.58	0.07
HDL-C, mmol/L	1.3 ± 0.32	1.3 ± 0.33	1.3 ± 0.31	0.70
TG/HDL-C ratio	0.84 ± 0.60	0.78 ± 0.50	0.89 ± 0.69	0.22

Values are presented as arithmetic mean ± standard deviation. *P* values were obtained with two-sided *t*-tests

Information on fasting plasma glucose and insulin was missing for 1 participant, on HOMA2-IR for 3 participants, and on TG, HDL-C and TG/HDL-C ratio for 91 participants

*BMI* body mass index; *DBP* diastolic blood pressure; *HDL-C* high-density lipoprotein cholesterol; *HOMA2-IR* homeostatic model assessment 2 of insulin resistance; *SBP* systolic blood pressure; *TG* triglycerides; *WC* waist circumference

**Fig. 1:**
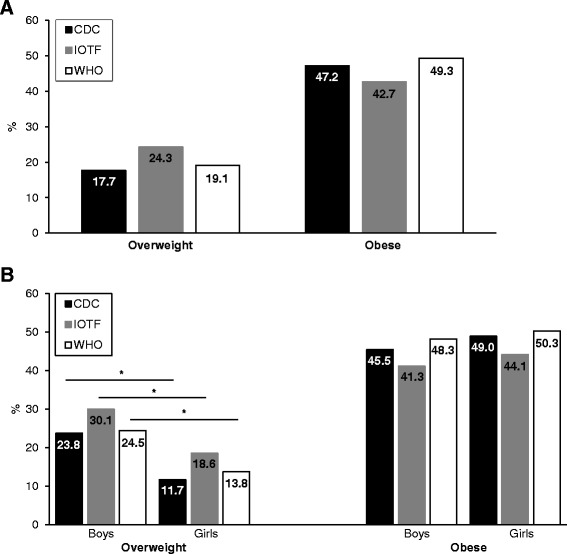
Overweight and obesity prevalence (**a**): whole study sample, (**b**): by gender) according to IOTF, CDC, and WHO classification systems among youth from Eeyou Istchee communities of northern Quebec, Canada, 2005–2009. Values are presented as prevalence (%). *Statistically different from girls (*P* < 0.05). CDC, Centers for Disease Control and Prevention; IOTF, International Obesity Task Force; WHO, World Health Organization

Prevalence estimates of overweight and obesity according to each BMI classification system are presented for the whole study sample (Fig. 1a) and by gender (Fig. 1b). Overall overweight prevalence was 24.3% with IOTF, 17.7% with CDC, and 19.1% with WHO criteria. The percentages of youth categorized as obese were 42.7%, 47.2%, and 49.3% respectively per IOTF, CDC, and WHO criteria. Overweight prevalence estimates were significantly higher (*P* < 0.05) among boys (11.5% with IOTF, 12.1% with CDC, and 10.7% with WHO) than girls. Overweight prevalence rates appeared to be greater with IOTF criteria regardless of gender, whereas CDC and WHO proportions were similar in boys and girls. Higher estimates of obesity were obtained for both genders with WHO and CDC systems compared to IOTF. However, these differences were not statistically significant. Level of agreement between weight status categories based on the three BMI classification systems are reported in Table 2. κw coefficients indicated almost perfect agreement between IOTF and CDC (κw = 0.93, 95% CI: 0.90–0.96; 93.41% agreement), IOTF and WHO (κw = 0.91, 95% CI: 0.87–0.94; 91.32% agreement), and WHO and CDC (κw = 0.94, 95% CI: 0.91–0.97; 94.45% agreement).

**Table 2:** Agreements between weight status based on IOTF, CDC and WHO classification systems

	CDC			
IOTF	Normal weight	Overweight	Obese	Total
Normal weight	95 (32.99%)	0	0	95
Overweight	6 (2.08%)	51 (17.71%)	13 (4.51%)	70
Obese	0	0	123 (42.71%)	123
Total	101	51	136	288
	WHO			
IOTF	Normal weight	Overweight	Obese	Total
Normal weight	90 (31.25%)	5 (1.74%)	0	95
Overweight	1 (0.35%)	50 (17.36%)	19 (6.60%)	70
Obese	0	0	123 (42.71%)	123
Total	91	55	142	288
	CDC			
WHO	Normal weight	Overweight	Obese	Total
Normal weight	91 (31.60%)	0	0	91
Overweight	10 (3.47%)	45 (15.63%)	0	55
Obese	0	6 (2.08%)	136 (47.22%)	142
Total	101	51	136	288

% of agreement = 91.32%; κw = 0.91, 95% CI: 0.87–0.94; *P* < 0.001

% of agreement = 94.45%; κw = 0.94, 95% CI: 0.91–0.97; *P* < 0.001

% of agreement was calculated by adding concordant percentages

*CDC* Centers for Disease Control and Prevention; *CI* confidence interval; *IOTF* International Obesity Task Force; *κw* weighted Kappa; *WHO* World Health Organization

Means of body fat and cardiometabolic risk factors by weight status and BMI classification systems are enumerated in Table 3. Body fat percentages were significantly higher from normal weight to obesity for IOTF, CDC, and WHO criteria (all *P*_trend_ < 0.001). All cardiometabolic risk factors except HDL-C presented the same pattern. SBP, DBP, fasting plasma glucose, fasting plasma insulin, HOMA2-IR, TG and TG/HDL-C ratio were significantly higher with weight status shift escalation, regardless of BMI system (all *P*_trend_ < 0.05), whereas HDL-C concentrations were significantly lower from normal weight to obesity (*P*_trend_ < 0.001).

**Table 3:** Means of body fat percentage and cardiometabolic risk factors according to IOTF, CDC and WHO classification systems

	Normal weight	Overweight	Obese	*P*_*trend*_
	IOTF			
	(*n* = 95)	(*n* = 70)	(*n* = 123)	
Body fat, %	22.8 ± 7.5^1^	29.7 ± 7.1^2^	41.7 ± 6.7^3^	< 0.001
SBP, mm Hg	102 ± 12.8^1^	103 ± 10.6^1^	108 ± 11.6^2^	< 0.001
DBP, mm Hg	60 ± 9.8^1^	61 ± 8.7^1^	65 ± 11.6^2^	< 0.001
Fasting plasma glucose, mmol/L	5.0 ± 0.40^1^	5.1 ± 0.35^1,2^	5.2 ± 0.50^2^	0.005
Fasting plasma insulin, pmol/L	86.4 ± 28.5^1^	116 ± 62.8^1^	207 ± 135^2^	< 0.001
HOMA2-IR	1.6 ± 0.52^1^	2.1 ± 1.1^1^	3.5 ± 2.0^2^	< 0.001
TG, mmol/L	0.76 ± 0.28^1^	0.90 ± 0.46^1^	1.1 ± 0.61^2^	< 0.001
HDL-C, mmol/L	1.5 ± 0.26^1^	1.3 ± 0.32^2^	1.1 ± 0.28^3^	< 0.001
TG/HDL-C ratio	0.55 ± 0.28^1^	0.7 ± 0.43^1^	1.1 ± 0.72^2^	< 0.001
	CDC			
	(*n* = 101)	(*n* = 51)	(*n* = 136)	
Body fat, %	23.2 ± 7.4^1^	28.5 ± 7.1^2^	41.0 ± 7.0^3^	< 0.001
SBP, mm Hg	102 ± 12.6^1^	102 ± 10.9^1^	108 ± 11.5^2^	< 0.001
DBP, mm Hg	60 ± 9.8^1^	60 ± 8.4^1^	65 ± 11.3^2^	< 0.001
Fasting plasma glucose, mmol/L	5.0 ± 0.39^1^	5.1 ± 0.35^1, 2^	5.2 ± 0.50^2^	0.002
Fasting plasma insulin, pmol/L	86.3 ± 28.5^1^	114 ± 67.0^1^	201 ± 131^2^	< 0.001
HOMA2-IR	1.6 ± 0.52^1^	2.1 ± 1.2^1^	3.5 ± 1.9^2^	< 0.001
TG, mmol/L	0.77 ± 0.28^1^	0.87 ± 0.48^1^	1.1 ± 0.60^2^	< 0.001
HDL-C, mmol/L	1.5 ± 0.26^1^	1.3 ± 0.34^1^	1.1 ± 0.29^2^	< 0.001
TG/HDL-C ratio	0.56 ± 0.27^1^	0.70 ± 0.44^1^	1.1 ± 0.71^2^	< 0.001
	WHO			
	(*n* = 91)	(*n* = 55)	(*n* = 142)	
Body fat, %	22.8 ± 7.5^1^	28.0 ± 6.9^2^	40.6 ± 7.2^3^	< 0.001
SBP, mm Hg	103 ± 13.0^1^	102 ± 10.9^1^	107 ± 11.4^2^	0.001
DBP, mm Hg	59 ± 9.9^1^	60 ± 7.9^1^	65 ± 11.4^2^	< 0.001
Fasting plasma glucose, mmol/L	5.0 ± 0.40^1^	5.0 ± 0.35^1,2^	5.2 ± 0.49^2^	0.008
Fasting plasma insulin, pmol/L	85.8 ± 28.0^1^	109 ± 62.9^1^	197 ± 130^2^	< 0.001
HOMA2-IR	1.6 ± 0.51^1^	2.0 ± 1.1^1^	3.4 ± 1.9^2^	< 0.001
TG, mmol/L	0.77 ± 0.29^1^	0.86 ± 0.46^1^	1.1 ± 0.59^2^	< 0.001
HDL-C, mmol/L	1.5 ± 0.27^1^	1.3 ± 0.32^1^	1.2 ± 0.30^2^	< 0.001
TG/HDL-C ratio	0.55 ± 0.28^1^	0.70 ± 0.43^1^	1.1 ± 0.70^2^	< 0.001

Values are presented as arithmetic means ± standard deviations. Means across weight status categories were compared by Scheffe’s tests, and *P*_trend_ was assessed by linear contrast

Information on fasting plasma glucose and insulin was missing for 1 participant, on HOMA2-IR for 3 participants and on TG, HDL-C and TG/HDL-C ratio for 91 participants

*CDC* Centers for Disease Control and Prevention; *DBP* diastolic blood pressure; *HDL-C* high-density lipoprotein cholesterol; *HOMA2-IR* homeostatic model assessment 2 of insulin resistance; *IOTF* International Obesity Task Force; *SBP* systolic blood pressure; *TG* triglycerides; *WC* waist circumference; *WHO* World Health Organization

^1,^ ^2,^ ^3,^Values with different superscript numbers are statistically different (*P* < 0.05)

Table 2 depicts mean differences of body fat and cardiometabolic risk factors in non-agreement youth (i.e., those considered overweight by IOTF, but obese by CDC or WHO criteria) compared to agreement youth (i.e., those classified as obese by both IOTF and CDC or IOTF and WHO cut-offs). Non-agreement youth exhibited significantly lower means (*P* < 0.05) of body fat, fasting plasma insulin and HOMA2-IR score than agreement youth. Non-agreement youth with WHO cut-offs also had significantly higher (*P* < 0.05) HDL-C levels than agreement youth.

## Discussion

We observed high prevalence rates of overweight and obesity regardless of the growth reference used. WHO cut-offs generated the highest prevalence estimates of overweight and obesity for participants overall and for both genders compared to CDC and IOTF. Despite a good level of agreement observed between these BMI classification systems using the weighted kappa statistic, we noted significant differences for discordant obesity. Youth, who were considered overweight by IOTF classification but not by CDC or WHO (non-agreement), exhibited less severe clinical obesity – characterized by lower levels of body fat, insulin and HOMA2-IR score.

Several studies, using these three classification systems, have reported inconsistent prevalence estimates of overweight and obesity among youth worldwide [13, 20–26]. Overall, WHO criteria yielded the highest overweight and obesity prevalence. We noted overweight/obesity prevalence of 67.0% with IOTF, 64.9% with CDC, and 68.4% with WHO criteria. Among Canadian youth, prevalence for the combined overweight/obesity category (5–17 years) was estimated to be 24.8% (8.4% obesity) with IOTF and 31.5% (11.7% obesity) with WHO [27]. Our results are similar to those of our previous study of Inuit youth from Nunavik [14]. Lower prevalence estimates of obesity were observed with IOTF and CDC compared to WHO criteria, which generated higher values regardless of gender. In other studies where IOTF generated the lowest estimates of both overweight and obesity prevalence [10–13], a higher prevalence of overweight was apparent for participants overall and by gender with IOTF compared to the CDC and WHO systems.

To investigate the accuracy of these three BMI classification systems in our population and in the absence of obesity-related outcomes (Table 4), we used body fat and different cardiometabolic risk factors [28] as surrogates. All variables were significantly higher whereas HDL-C levels were significantly lower from normal weight to obesity regardless of the BMI classification system. Our previous results on Inuit youth disclosed similar patterns for plasma insulin and high-sensitivity C-reactive protein concentrations for the three growth references. Adiponectin levels were also significantly higher but only with WHO classification.

**Table 4:** Mean difference of body fat percentage and cardiometabolic risk factors according to agreement and non-agreement between weight status based on IOTF and CDC, and IOTF and WHO classification systems

	IOTF vs. CDC			
	Non-agreement IOTF overweight/ CDC obese (*n* = 13)	Agreement IOTF obese/ CDC obese (*n* = 123)	Mean difference (95% CI)	*P*
Body fat, %	34.5 ± 6.6	41.7 ± 6.7	−7.1 (−11.0, −3.2)	<0.001
SBP, mm Hg	104 ± 10.4	108 ± 11.6	−3.5 (−10.2, 3.1)	0.29
DBP, mm Hg	63 ± 8.9	65 ± 11.6	−2.1 (−8.7, 4.4)	0.52
Fasting plasma glucose, mmol/L	5.0 ± 0.40	5.2 ± 0.50	−0.17 (−0.45, 0.12)	0.26
Fasting plasma insulin, pmol/L	142 ± 49	207 ± 135	−65.3 (−102, −28.5)	0.001
HOMA2-IR	2.6 ± 0.86	3.5 ± 2.0	−0.97 (−1.6, −0.36)	0.003
TG, mmol/L	1.1 ± 0.45	1.1 ± 0.61	0.003 (−0.50, 0.51)	0.99
HDL-C, mmol/L	1.2 ± 0.32	1.1 ± 0.28	0.11 (−0.13, 0.35)	0.38
TG/HDL-C ratio	0.97 ± 0.46	1.1 ± 0.72	−0.14 (−0.74, 0.46)	0.64
	IOTF vs. WHO			
	Non-agreement IOTF overweight/ WHO obese (*n* = 19)	Agreement IOTF obese/ WHO obese (*n* = 123)	Mean difference (95% CI)	*P*
Body fat, %	33.6 ± 6.5	41.7 ± 6.7	−8.1 (−11.3, −4.8)	<0.001
SBP, mm Hg	104 ± 9.3	108 ± 11.6	−4.2 (−9.7, 1.3)	0.13
DBP, mm Hg	63 ± 10.1	65 ± 11.6	−2.2 (−7.7, 3.4)	0.44
Fasting plasma glucose, mmol/L	5.1 ± 0.39	5.2 ± 0.50	−0.12 (−0.36, 0.12)	0.32
Fasting plasma insulin, pmol/L	135 ± 55.3	207 ± 135	−71.9 (−107, −36.6)	<0.001
HOMA2-IR	2.5 ± 0.98	3.5 ± 2.0	−1.1 (−1.7, −0.50)	<0.001
TG, mmol/L	0.97 ± 0.40	1.1 ± 0.61	−0.18 (−0.55, 0.20)	0.36
HDL-C, mmol/L	1.4 ± 0.35	1.1 ± 0.28	0.23 (0.04, 0.42)	0.02
TG/HDL-C ratio	0.77 ± 0.43	1.1 ± 0.72	−0.34 (−0.78, 0.10)	0.13

Values are presented as arithmetic means ± standard deviations. *P* values were obtained by *t*-tests

Information on fasting plasma glucose and insulin was missing for 1 participant, on HOMA2-IR for 3 participants, and on TG, HDL-C and TG/HDL-C ratio for 91 participants

*CDC* Centers for Disease Control and Prevention; *DBP* diastolic blood pressure; *HDL-C* high-density lipoprotein cholesterol; *HOMA2-IR* homeostatic model assessment 2 for insulin resistance; *IOTF* International Obesity Task Force; *SBP* systolic blood pressure; *TG* triglycerides; *WHO* World Health Organization

In our sample, IOTF criteria appear to be more accurate than CDC or WHO in identifying youth with obesity. According to the IOTF classification system, 90.4% of cases with obesity would be classified as obese with CDC and 86.6% with WHO. Also, we observed that youth classified as overweight by IOTF but not by CDC or WHO exhibited less severe clinical obesity. The lower proportion of subjects with obesity identified by IOTF was reflected by higher classification in the overweight category compared to CDC or WHO. In other words, false-positive subjects with obesity identified by CDC or WHO criteria were adequately classified as overweight by IOTF.

Our study has some limitations. Because of limitations inherent in cross-sectional investigations, no causal relationship can be ascertained. The cross-sectional design did not provide information on weight status variations through age and timing growth patterns. As the study participants were Indigenous, with a high prevalence of overweight and obesity, generalizability of the observed associations may be limited to similar populations. The strengths of this study are its relatively large sample and direct assessment of weight, height, body fat and large number of biological measurements under fasting conditions, especially cardiometabolic risk factors. These allowed us to investigate the accuracy of the three BMI classification systems to estimate overweight and obesity prevalence in our population.

## Conclusions

In summary, IOTF criteria seem to be more suitable than CDC and WHO in identifying more severe clinical obesity in our sample of Cree youth of Eeyou Istchee, northern Quebec. IOTF criteria generated lower obesity prevalence estimates than CDC and WHO, but all three classification systems were associated with increasing body fat and cardiometabolic risk factors from normal weight to obesity. The persistence of childhood obesity in adulthood [29, 30] highlights the need for an early identification of excess weight onset. A consensus on childhood obesity assessment is essential.

## Abbreviations

BMI: Body mass index
CBHSSJB: Cree Board of Health and Social Services of James Bay
CDC: Centers for Disease Control and Prevention
DBP: Diastolic blood pressure
HDL-C: High-density lipoprotein cholesterol
HOMA2-IR: Homeostatic model assessment 2 of insulin resistance
IOTF: International Obesity Task Force
SBP: Systolic blood pressure
TG: Triglycerides
WC: Waist circumference
WHO: World Health Organization
Κw: Weighted kappa
